# Small non-coding RNA landscape of extracellular vesicles from human stem cells

**DOI:** 10.1038/s41598-018-33899-6

**Published:** 2018-10-19

**Authors:** Sippy Kaur, Ahmed G. Abu-Shahba, Riku O. Paananen, Heidi Hongisto, Hanna Hiidenmaa, Heli Skottman, Riitta Seppänen-Kaijansinkko, Bettina Mannerström

**Affiliations:** 1 Department of Oral and Maxillofacial Diseases, University of Helsinki and Helsinki University Hospital, Helsinki, Finland; 2 Faculty of Medicine and Life Sciences, BioMediTech, University of Tampere, Tampere, Finland; 3 Helsinki Eye Lab, Ophthalmology, University of Helsinki and Helsinki University Hospital, Helsinki, Finland; 4 Department of Oral and Maxillofacial Surgery, Faculty of Dentistry, Tanta University, Tanta, Egypt

## Abstract

Extracellular vesicles (EVs) are reported to be involved in stem cell maintenance, self-renewal, and differentiation. Due to their bioactive cargoes influencing cell fate and function, interest in EVs in regenerative medicine has rapidly increased. EV-derived small non-coding RNA mimic the functions of the parent stem cells, regulating the maintenance and differentiation of stem cells, controlling the intercellular regulation of gene expression, and eventually affecting the cell fate. In this study, we used RNA sequencing to provide a comprehensive overview of the expression profiles of small non-coding transcripts carried by the EVs derived from human adipose tissue stromal/stem cells (AT-MSCs) and human pluripotent stem cells (hPSCs), both human embryonic stem cells (hESCs) and human induced pluripotent stem cells (hiPSC). Both hPSCs and AT-MSCs were characterized and their EVs were extracted using standard protocols. Small non-coding RNA sequencing from EVs showed that hPSCs and AT-MSCs showed distinct profiles, unique for each stem cell source. Interestingly, in hPSCs, most abundant miRNAs were from specific miRNA families regulating pluripotency, reprogramming and differentiation (miR-17-92, mir-200, miR-302/367, miR-371/373, CM19 microRNA cluster). For the AT-MSCs, the highly expressed miRNAs were found to be regulating osteogenesis (let-7/98, miR-10/100, miR-125, miR-196, miR-199, miR-615-3p, mir-22-3p, mir-24-3p, mir-27a-3p, mir-193b-5p, mir-195-3p). Additionally, abundant small nuclear and nucleolar RNA were detected in hPSCs, whereas Y- and tRNA were found in AT-MSCs. Identification of EV-miRNA and non-coding RNA signatures released by these stem cells will provide clues towards understanding their role in intracellular communication, and well as their roles in maintaining the stem cell niche.

## Introduction

Stem cells are responsible for the development and regeneration of tissues and maintaining steady-state of organ homeostasis. Stem cells of various types exist; pluripotent stem cells (PSCs), such as embryonic stem cells (ESCs) and induced pluripotent stem cells (iPSCs) have the potential to differentiate into all types of adult human tissues, while stem cells residing in the adult individual, such as mesenchymal stem/stromal cell (MSCs) have a more limited differentiation capacity^1^. Tissue development and regeneration involves cell activities such as recruitment, proliferation and differentiation, which are mediated by autocrine or paracrine effectors^2^. Therapeutic activities mediated by paracrine signalling in stem cells have been well documented.

The paracrine effectors of stem cells, such as extracellular vesicles (EVs), which mimic stem cell properties, could represent a relevant therapeutic option in regenerative medicine. EVs are important mediators of intercellular communication and regulate bidirectional transfer of proteins, lipids and nucleic acids between cells via specific receptor-mediated interactions^3^. The contribution of stem cell-derived EVs in lineage commitments, maintenance of self-renewal, differentiation, maturation, efficiency of cellular reprogramming and cell fate determination are largely regulated by non-coding RNA (ncRNA)^4^. Small ncRNA (<200 nucleotides) includes microRNA (miRNA), small nuclear RNA (snRNA), small nucleolar RNA (snoRNA), piwi-interacting RNA (piRNA), transfer RNA (tRNA), small ribosomal RNA (rRNA), and small cytoplasmic RNA (Y RNA). These are involved in various biological processes and maintain the equilibrium between pluripotency and differentiation in stem cells, thereby aiding in governing stem cell potency and lineage-specific fate decisions^5,6^. Furthermore, the ncRNAs are known to be sorted into EVs thus modulating cellular processes^7,8^. Therefore, EV-derived ncRNAs are potential mediators of paracrine effects of stem cells.

Small ncRNAs, particularly microRNAs (miRNAs) which are central to gene regulation and cellular fate determination, can also mediate their regulatory effects via EVs^9^. miRNAs are small endogenous non-coding RNAs that function as posttranscriptional regulators of gene expression through translational inhibition or by promoting the degradation of mRNA. They are important regulators of reprogramming processes, maintenance of pluripotency and differentiation of stem cells^10^. EV-derived miRNAs thereby are mediators of the extended paracrine effects of stem cells^11–13^. Thus, it could be concluded that intercellular communication mediated by transfer of EV-derived miRNAs coordinate the intercellular regulation of gene expression, which eventually affects the fate of the stem cells and their surrounding niches.

The primary goal of this study was to characterize the EV-derived miRNAs and other small ncRNAs of AT-MSCs and hPSCs cultured *in vitro*, and to explore their biological relevance. Identification of EV-miRNA and other small ncRNA signatures released by these stem cells will provide clues towards understanding the role of these extracellular RNAs in intercellular communication and their role in regulation of stemness *in vitro*.

Taken together, our data indicates that the miRNAs previously reported to be the important regulators of stem cells at cellular level are also present in their EVs, indicating a regulatory role that can be mediated via EVs. Therefore, transfer of these miRNAs and small ncRNA to other cells may promote lineage commitment in stem cells, and subsequently enhance differentiation in a cell therapy setting.

## Results

### hPSCs and AT-MSCs showed characteristics typical of respective stem cell type

Both stem cell types (MSC and PSC) were characterized with standard methods for the respective cell type in conjunction with media collections for EV extraction. The cells showed characteristic morphology, marker expression, and differentiation capacity.

**Figure 1:**
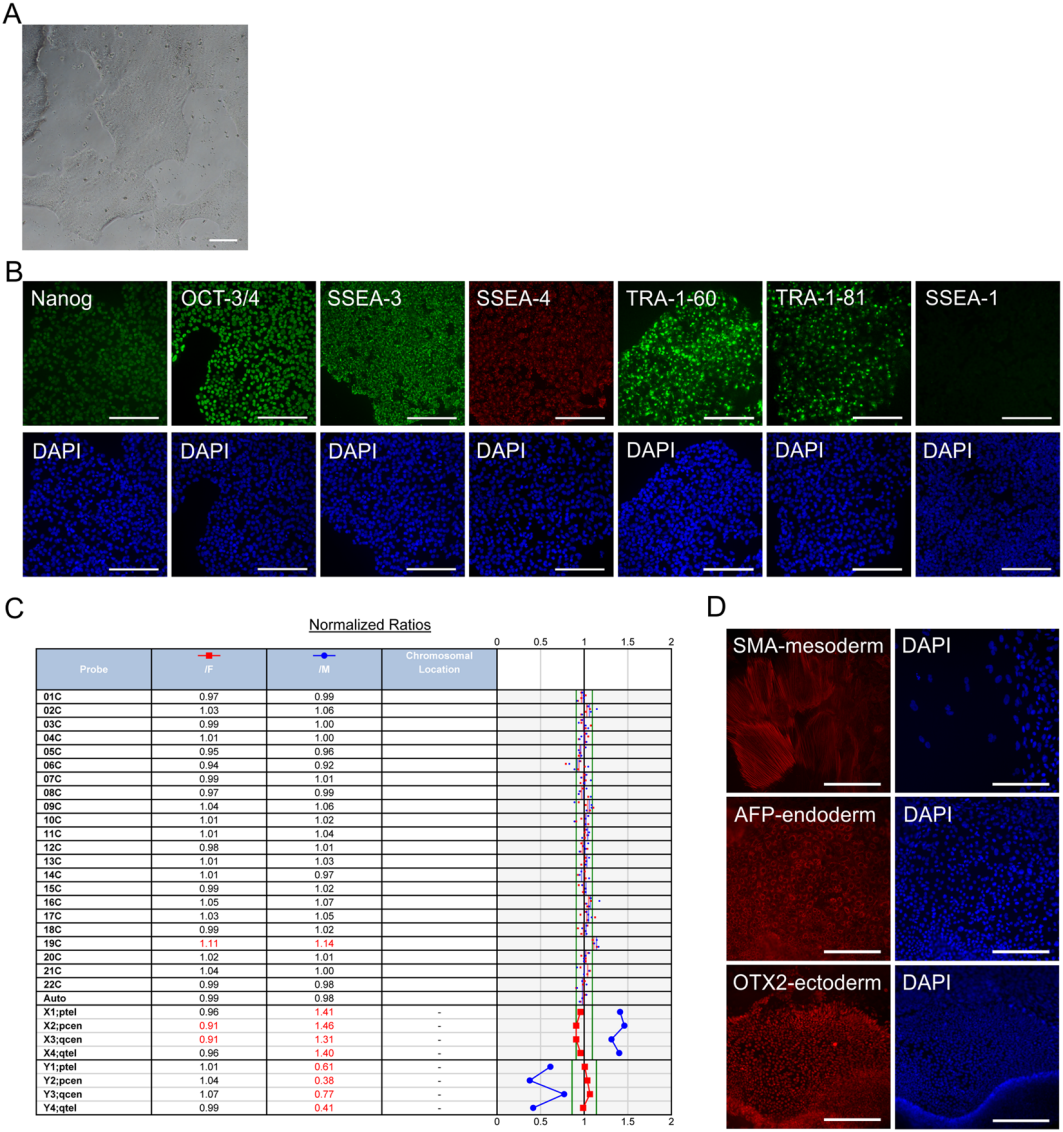
PSC characterisation. (**A**) Human PSC-1 characterized for A) typical undifferentiated colony morphology in phase contrast image and (**B**) expression of pluripotency markers Nanog, OCT-3/4, SSEA-3, SSEA-4, TRA-1-60, and TRA-1-81, and lack of expression of early differentiation marker SSEA-1 after immunofluorescence staining. Corresponding nuclei stains with DAPI shown. (**C**) Cells showed normal female (46, XX) karyotype after 28 passages in total (9 passages in feeder-free culture). The results of the KaryoLite BoBs assay are shown as signal relative to karyotypically normal female (/F, red) and male (/M, blue) genomic DNA used as a reference (equal to 1) for each of the probes covering both p and q arms of all chromosomes. Software threshold for changes shown as a green lines and deviations in red. (**D**) Pluripotency shown after spontaneous differentiation *in vitro* as expression of markers for mesoderm, endoderm, and ectoderm. All scale bars 200 µm.

Human PSCs grew as well-defined colonies (Fig. 1A) and further as smooth monolayers reaching confluence, typical to undifferentiated hPSCs on laminin-521. The cells showed uniform expression of core pluripotency transcription factors Nanog, and octamer-binding transcription factor-3/4 (OCT-3/4), as well as typical hPSC surface marker expression of stage-specific embryonic antigens (SSEA) -3 and -4, tumor rejection antigens (TRA)-1-60 and -1-81, as well as lack of expression of early differentiation marker SSEA-1 (Fig. 1B). Further, the cells showed normal diploid karyotypes (Fig. 1C). Pluripotency was confirmed *in vitro* as differentiation capacity to derivative cells of all three embryonic germ layers (Fig. 1D). Characterisation of the hPSC-1 line is shown in Fig. 1 and hPSC2 line in Supplemental 1.

**Figure 2:**
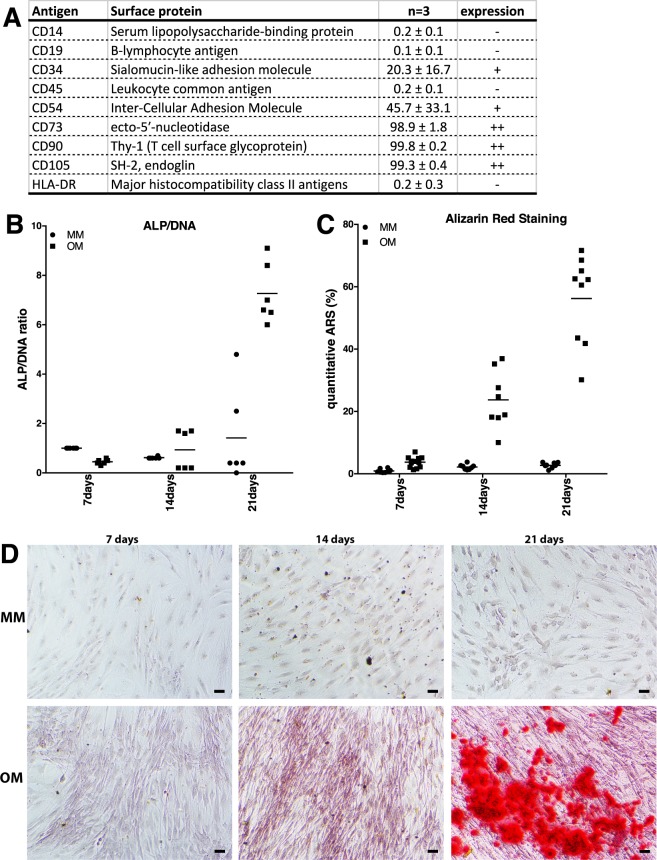
AT-MSC characterisation. Surface marker expression (%) of undifferentiated AT-MSCs (n = 3) analyzed by flow cytometric analysis (**A**). Quantitative data of early and later phase response of osteogenesis by (**B**) alkaline phosphatase activity (early response) and (**C**) Alizarin red staining (late response). (**D**) Alizarin red staining for assessment of mineralized matrix formation. Results show no detection of mineralized matrix in control condition (MM), while osteogenically induced cultures (OM) show enhanced mineralized matrix formation after 21 days. Dots represent biological and technical replicates, and bars represent means. MM; maintenance medium, OM; osteogenic medium, ARS; alizarin red staining, ALP; alkaline phosphatase. Scale bar 100 μm.

The characterization of AT-MSCs conformed with previous results^14,15^ and the standards defined by the International Society for Cellular Therapy (ISCT)^16^, by cells being successfully isolated and expanded as adherent fibroblast-like cells. Further, the AT-MSCs cultured in FBS-supplemented medium expressed surface markers CD54, CD73, CD90 and CD105, while lacking the expression of hematopoietic markers CD14, CD19, CD45 and HLA-DR (Fig. 2A). In addition, AT-MSCs showed moderate expression of CD34, as previously reported for AT-MSCs^14,15,17^. Furthermore, CD34 and CD54 showed donor variability^18^.

The osteogenic differentiation capacity was analyzed for the AT-MSC donor cell lines, assessing both early differentiation capacity by alkaline phosphatase activity and later phase by alizarin red staining for mineralized matrix formation. The early response showed that the osteogenic differentiation capacity gradually increased up to 21 days in osteogenic induction cultures (OM), while control conditions (MM) showed low or no response (Fig. 2B). The alkaline phosphatase activity was normalized to the total DNA. Similarly, the later phase response in terms of mineralized matrix formation, showed gradual increase of matrix accumulation up to 21 days in OM, with no matrix formation seen in control conditions (Fig. 2C,D).

### hPSCs and AT-MSCs showed robust secretion of EVs with a cell type specific size distribution

**Figure 3:**
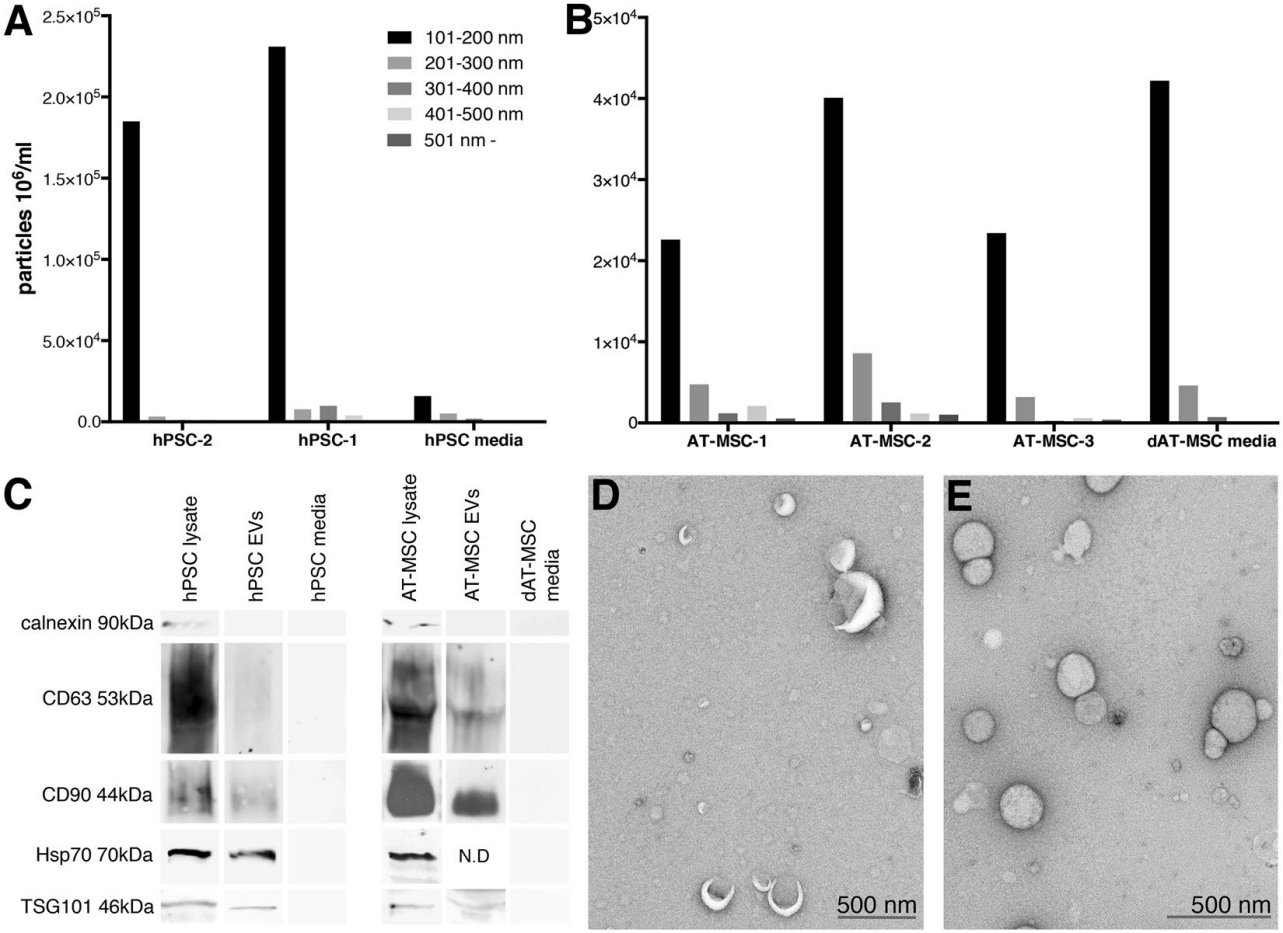
EV characterisation. (**A**,**B**) EV concentration and size distribution measured by nanoparticle tracking analysis (NTA). On y-axis concentrations (particles 10^6^/ml) are shown and on x-axis EV samples of hPSC, AT-MSC and their respective medias; hPSC media, and dAT-MSC media (EV-depleted AT-MSC media). In general. hPSCs contained more particles than AT-MSCs. (**C**) Western blotting showed presence of stronger signals of EV markers CD63 and CD90 in AT-MSC EVs as compared to hPSC EVs. TSG101 expression was seen in both hPSC and AT-MSC EVs. hPSC EVs showed clear band of hps70, whereas Hsp70 signal was not detectable (N.D) in AT-MSC EVs due to technical reasons. Absence of Calnexin protein in all the EV samples indicated purity of the samples. (**D**,**E**) TEM images of hPSC and AT-MSCs. Scale bar 500 nm.

EVs were extracted from the culture media of AT-MSCs and hPSCs using ultracentrifugation^15^. Both cell types secreted abundant amount of EVs and their presence was confirmed by NTA, EM and WB. Characterization of EVs by NTA (Fig. 3A,B) supported the WB analysis with EV markers such as TSG101, Hsp70, CD63, CD90 (Fig. 3C). EM indicated the presence of heterogeneous population of EVs in both cell types (Fig. 3D,E). WB results indicated that AT-MSC EVs showed stronger signals of CD63 and CD90 than hPSC EVs. TSG101 expression was seen in both cell lines EVs. hPSC EVs showed a clear band of Hsp70, whereas due to technical reasons Hsp70 signal was not detectable (N.D) in AT-MSC EVs. Differences in the expression level of EV markers between cell types in our study could be explained by fact that the EV biogenesis pathways could be different among these cell lines. The purity of EVs was confirmed with the absence of the endoplasmic reticulum marker calnexin in all our EV samples. NTA results showed that AT-MSCs contained 2–4 × 10^4^ of the 100–200 nm sized particles as the major group with a distribution of other sizes in smaller quantities, while hPSCs secreted 1.8–2.3 × 10^5^ of 100–200 nm sized particles (101–200 nm) and very small quantities of larger particles. The media used for EV collections were analyzed parallel to samples. EV-depleted FBS used in AT-MSC culture media was analyzed by NTA displayed large number of particles (101–200 nm), hPSC medium on the other hand as expected contained insignificant amounts of EV sized particles. Particle size determination by NTA is not entirely accurate as it cannot differentiate EVs from other particles such as protein aggregates and lipoproteins^19^. This results in false positives and impairs the reliability of the analysis. Therefore a combination of TEM, which was also used in this study is needed to visualize and confirm the presence of vesicles.

### hPSCs and AT-MSCs EVs have unique miRNA signature

**Figure 4:**
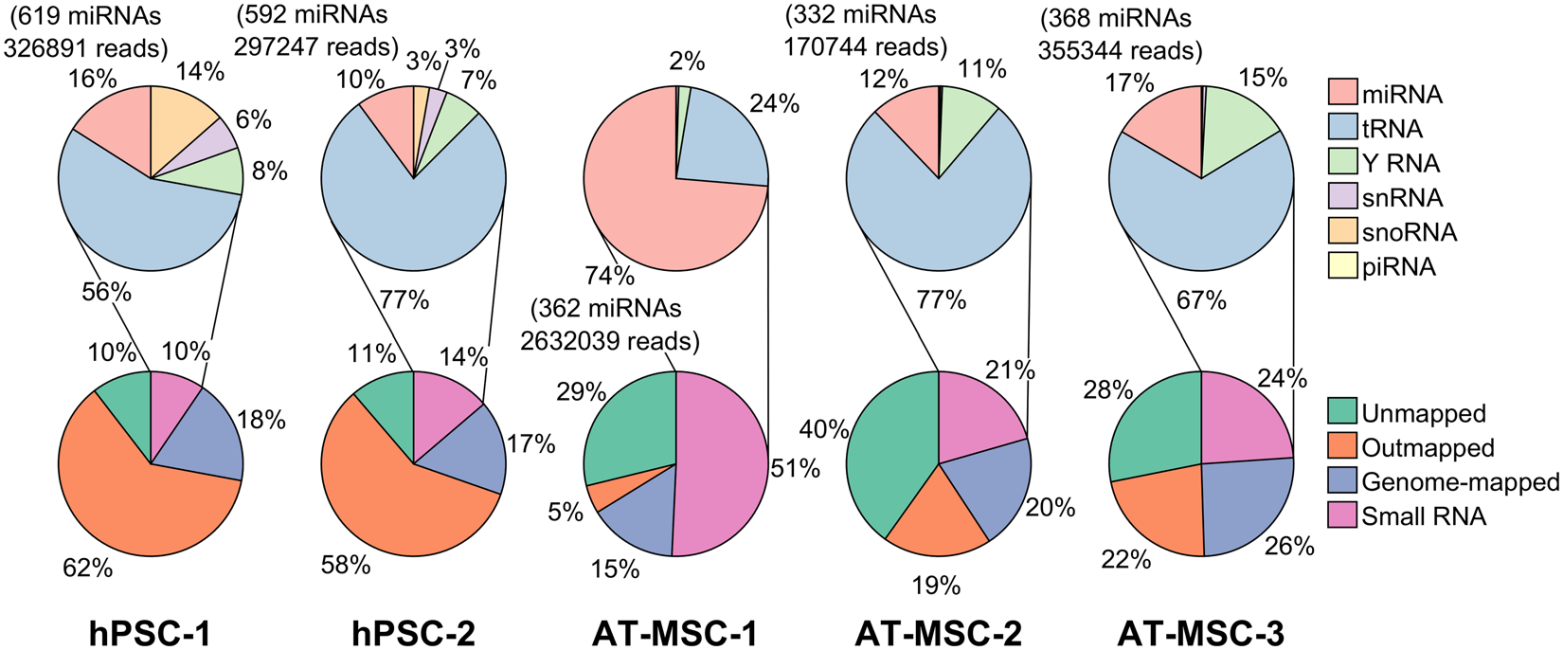
Mapping of sequencing reads. Relative composition of diverse RNA classes in hPSC and AT-MSC EVs. Only 1.4-1.5% and 2.5–37% of total reads mapped to miRNA in hPSC and AT-MSC, respectively.

Next generation sequencing was used to determine the small ncRNA expression profile of five EV samples (3 × AT-MSC and 2 × hPSC) as well as the corresponding media. Mapping of the reads from the sequencing is shown in Fig. 4, 71% and 43% of the reads of hPSC and AT-MSC samples, respectively, failed to align to the reference genome or were discarded, since they were outmapped, ie. mapped to abundant sequences such as polyA and polyC homopolymers, ribosomal RNA, the mitochondrial chromosome or ΦX174-genome. Most outmapped reads (>80% in all samples) were mapped to 28 S, 18 S, or 5.8 S ribosomal RNA. Out of the remaining reads, approximately half were mapped to small RNAs. On average, 13% of small RNA reads in hPSC samples were mapped to miRNA, while tRNA fragments (69%), Y RNA (7%), snRNA (4%), snoRNA (7%) and piRNA (<1%) made up the rest of reads. In AT-MSC samples, only miRNA (44%), tRNA (47%) and Y RNA (8%) were detected, while other small RNA types comprised <1% or total reads.

**Figure 5:**
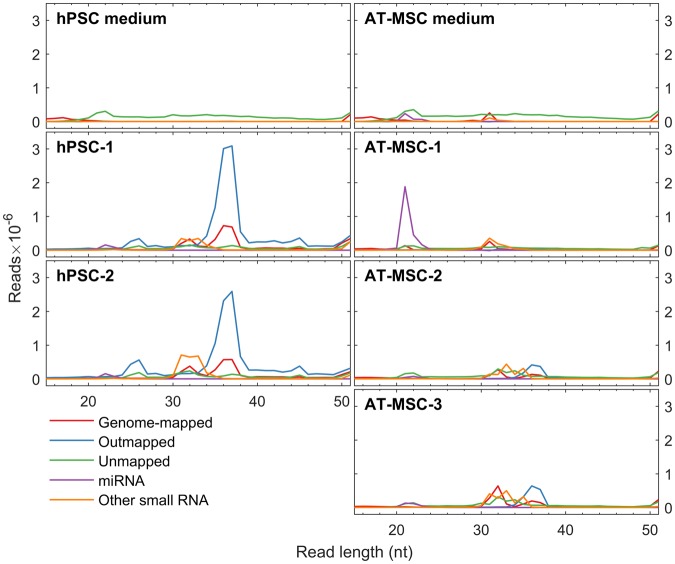
Sequencing reads length distribution. Length distribution of aligned reads from all 5 EV stem cell samples and their respective media.

Length distribution of reads is shown in Fig. 5. An average of 21.5 million and 8.5 million raw reads were obtained from hPSC and AT-MSC samples, respectively, whereas 7.2 million (hPSC) and 9.3 million (AT-MSC) reads were obtained from the corresponding media. AT-MSC medium showed similar levels of mapped reads in the miRNA (20–25 nucleotides) and other small RNA (30–35 nucleotides) ranges as AT-MSC samples, except for sample AT-MSC-1, which had very high levels of miRNA reads. In contrast, hPSC medium had very low number of reads in the miRNA and smRNA range, resulting in a lower background signal in the hPSC samples compared to AT-MSC samples. The higher read count of hPSC samples compared to AT-MSC samples is explained by a large peak of 35–40 nucleotide reads, which were mostly mapped to ribosomal RNA.

### hPSCs and AT-MSCs have distinct miRNA and small RNA profiles

**Figure 6:**
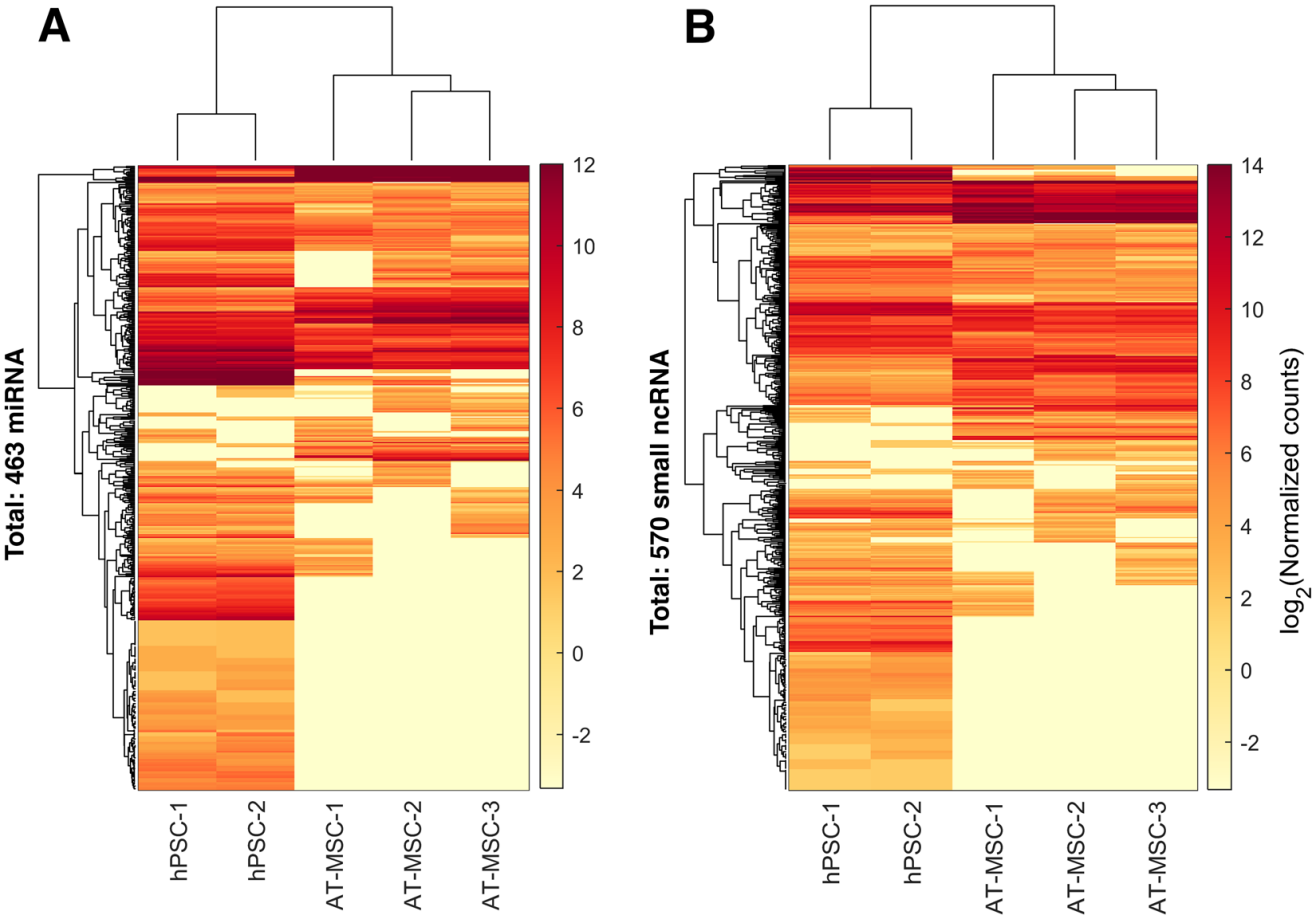
Heat map cluster analysis of EV-RNA. Unsupervised cluster analysis on AT-MSC and hPSC based on their EV-miRNAs (**A**) and ncRNAs (**B**). AT-MSCs and hPSCs clearly clustered separately based on both EV-derived miRNA and ncRNA.

Unsupervised clustering was performed on EV-miRNAs in order to assess the variability among the samples (Fig. 6A). hPSC and AT-MSC samples showed clearly different EV-miRNA expression patterns, but strong similarity within sample groups was observed. Expression patterns of AT-MSC-1 was distinctively different from the other two replicates. This could be due to the medical history of this donor (gastric bypass, antidepressant medication). hPSCs showed a wider range of expressed EV-miRNAs compared to AT-MSCs, with 159 out of 463 miRNAs only present in hPSC EVs. Unsupervised clustering analysis on EV-derived small ncRNA also separated AT-MSCs from hPSCs (Fig. 6B). SNORD family was exclusively present in hPSC-EVs, whereas Y and tRNA fragments were highly expressed in AT-MSCs EVs (Supplemental 2).

EV-miRNAs were divided into groups based on significant (p < 0.01) differential expression or expression in both sample types (|FC| < 2) and a cutoff of 32 mean counts per million (CPM) (Tables 1 and 2). AT-MSCs and hPSCs had unique miRNA profiles at their EV level. Finally, 32 EV-miRNA, majority of which are positive and negative regulators of osteogenic differentiation was exclusively present in MSC, whereas 77 miRNA involved in maintaining pluripotency (including mir families: mir-371/372, mir-302/367, 200, 17/92 and C19MC) were highly represented in hPSC.

**Table 1:** Differentially expressed major EV-miRNA families in hPSC with the corresponding normalized counts and p-values.

Family	Family members	log_2_FC	log_2_CPM	pValue	FDR	Major Functions in PSCs	Reference
mir 17-92	hsa-miR-17-5p	−4,34568	9,012717	0,000855	0,00184	Controls proliferation of embryonic stem cells	^94^
	hsa-miR-18a-5p	−4,12927	5,365265	0,002687	0,005021		
	hsa-miR-20a-5p	−3,39956	9,942874	5,93E-05	0,000159		
	hsa-miR-92a-3p	−2,28835	14,64776	0,005145	0,009065		
mir-200	hsa-miR-200a-3p	−4,18521	6,916635	0,005677	0,009636	regulates differentiation, Maintaining pluripotency and regulates epithelial-mesenchcymal transition	^95^
	hsa-miR-200a-5p	−4,53501	5,812451	0,000615	0,001422		
	hsa-miR-200b-3p	−6,81357	10,64906	1,04E-06	4,1E-06		
	hsa-miR-429	−9,26846	6,001451	4,62E-08	2,59E-07		
mir-302/367	hsa-miR-302a-3p	−15,6675	12,33651	9,32E-25	1,3E-22	Maintaining pluripotency	^42^
	hsa-miR-302a-5p	−13,4935	17,28532	5,1E-20	1,89E-18		
	hsa-miR-302b-3p	−13,5506	16,09634	9,86E-18	3,04E-16		
	hsa-miR-302c-3p	−15,5738	12,24285	1,41E-24	1,3E-22		
	hsa-miR-302c-5p	−11,0944	9,555795	6,18E-16	1,27E-14		
	hsa-miR-302d-3p	−13,7161	12,16085	4,98E-23	3,07E-21		
	hsa-miR-302d-5p	−8,71179	5,475559	5,61E-07	2,36E-06		
	hsa-miR-367-3p	−9,95691	6,665547	2,02E-09	1,29E-08		
mir-371/373	hsa-miR-371a-3p	−8,62254	5,393327	1E-06	4,03E-06	regulating cell proliferation, differentiation	^35^
	hsa-miR-371a-5p	−11,2134	7,898332	1,21E-12	1,32E-11	and reprogramming	
	hsa-miR-372-3p	−13,3871	11,83298	3,78E-22	1,75E-20		
	hsa-miR-373-3p	−9,00191	5,748367	1,49E-07	7,63E-07		
C19MC	hsa-miR-516a-5p	−8,51958	5,296551	2,2E-06	7,52E-06	maintaining stem cell self-renewal and pluripotency	^96^
	hsa-miR-516b-5p	−9,69778	8,987653	1,64E-13	2,09E-12		
	hsa-miR-517a-3p	−9,14288	5,88151	9,03E-08	4,78E-07		
	hsa-miR-517b-3p	−8,77055	5,528605	1,4E-06	5,17E-06		
	hsa-miR-518b	−10,5662	7,260425	5,46E-11	4,39E-10		
	hsa-miR-520c-3p	−8,57565	5,348277	1,68E-06	5,87E-06		
	hsa-miR-520f-3p	−10,0952	6,799461	1,09E-09	7,46E-09		
	hsa-miR-520g-3p	−8,2576	6,709774	1,25E-08	7,47E-08		
	hsa-miR-522-3p	−8,44063	5,225189	2,99E-06	1,01E-05		
	hsa-miR-523-3p	−8,20928	5,013337	1,17E-05	3,49E-05		
	hsa-miR-526b-5p	−8,8302	5,585032	7,1E-07	2,92E-06		
	hsa-miR-1323	−11,8527	8,531433	1,69E-13	2,09E-12

**Table 2:** Differentially expressed major EV-miRNA families in AT-MSC with the corresponding normalized counts and p-values.

Family	Family members	log_2_FC	log_2_CPM	pValue	FDR	Major Functions in MSCs	References
let-7/mir-98	hsa-let-7a-3p	9,151431	6,477715	1,41E-05	4,15E-05	Regulates differentiation	^53, 54, 64^
	hsa-let-7a-5p	4,438344	13,48553	5,38E-06	1,72E-05		
	hsa-let-7b-3p	9,306488	6,621799	5,87E-05	0,000159		
	hsa-let-7b-5p	9,666006	14,19212	5,27E-13	6,09E-12		
	hsa-let-7c-5p	6,377311	10,45171	5,57E-09	3,43E-08		
	hsa-let-7e-5p	5,912685	9,64524	2,31E-07	1,15E-06		
	hsa-let-7f-5p	6,292228	12,90261	1,65E-09	1,09E-08		
	hsa-let-7i-5p	8,040472	14,0149	9,88E-12	9,62E-11		
	hsa-miR-98-5p	10,92755	8,203416	1,92E-08	1,11E-07		
mir-10/100	hsa-miR-10a-5p	7,995657	13,27917	2,67E-10	1,9E-09		
	hsa-miR-10b-3p	7,559436	5,017372	0,001355	0,002849		
	hsa-miR-10b-5p	8,498145	14,36205	8,18E-12	8,41E-11		
	hsa-miR-100-5p	4,009269	13,98291	0,000211	0,000519	negative regulator of osteogenic differentiation	^62^
mir-125	hsa-miR-125a-5p	3,250007	10,45281	0,000975	0,002073		
	hsa-miR-125b-1-3p	5,844325	8,026384	0,004338	0,007792	Suppress osteogenesis	^63, 97^
	hsa-miR-125b-5p	6,371527	9,723062	3,62E-07	1,67E-06		
mir-196	hsa-miR-196a-5p	7,26496	7,494054	0,001717	0,003378	Promotes osteogenesis	^60^
	hsa-miR-196b-5p	7,761247	5,191769	0,00082	0,001792		
mir-199	hsa-miR-199a-3p	3,994405	8,941022	0,000276	0,000671	Involved in osteogenesis/chondogenesis	^98^
	hsa-miR-199a-5p	5,906115	9,147113	3,88E-06	1,28E-05		
	hsa-miR-199b-3p	3,924898	8,599356	0,000403	0,000968		
	hsa-miR-199b-5p	3,021359	7,643468	0,003009	0,005567	positive role in osteoblast differentiation	^56^
mir-148/152	hsa-miR-152-3p	4,293438	9,473328	4,73E-05	0,000131		
	hsa-miR-22-3p	4,552346	10,88709	5,96E-06	1,84E-05	regulating balance in osteogenesis/Adipogenesis	^58^
	hsa-miR-24-3p	2,867598	10,70777	0,001581	0,003249	inhibits osteogenesis	^66^
	hsa-miR-27a-3p	2,971421	11,04311	0,001444	0,003001	inhibits osteogenesis	^66, 99^
	hsa-miR-143-3p	6,997134	13,46121	1,9E-11	1,64E-10		
	hsa-miR-144-3p	10,80517	8,085471	1,68E-06	5,87E-06		
	hsa-miR-193b-5p	4,056847	6,250916	0,002251	0,004294	Regulate chondrogenesis	^100^
	hsa-miR-195-3p	3,357619	6,148953	0,004834	0,008599	negative regulator of osteogenesis	^65^
	hsa-miR-542-3p	4,172502	5,528398	0,005462	0,009455		
	hsa-miR-615-3p	9,77289	10,03215	2,62E-05	7,36E-05	negative regulator of osteogenesis	^67^

### Comparison of miRNA and other small RNAs detected in hPSC and AT-MSC medias

**Figure 7:**
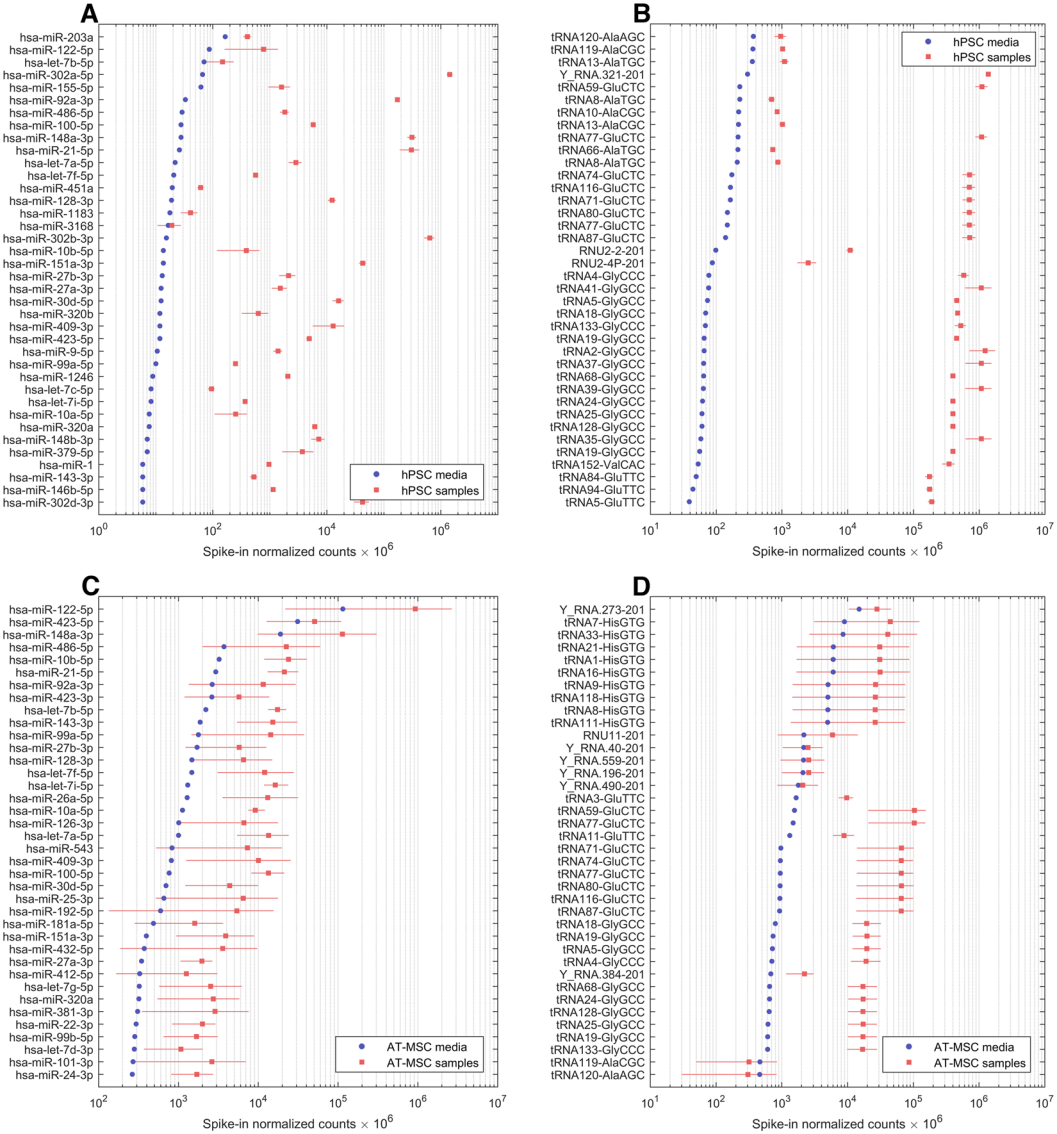
Comparison of sample miRNA and small RNA to the respective media. Read counts normalized to external spike-ins for 38 miRNAs and other small RNAs with highest counts in each medium (blue) and mean counts in corresponding samples (red). Error bars depict the range of reads (min-max). miRNA and small ncRNA identified in hPSC media (**A**,**B**) and AT-MSC media (**C**,**D**).

Due to the relatively high number of reads obtained from the AT-MSC medium, we compared each type of samples to their respective media. Various normalization strategies are used for EV-RNA data, such as library size, geometric mean, endogenous references, or external spike-ins^20^. Since practically no similarity in RNA content can be assumed to exist between the unconditioned media and cell-derived EV samples, we normalized the reads to external spike-ins, as has been done previously to compare RNAs between different types of samples^8^. As can be seen in Fig. 7, considerable amounts of both miRNAs and other small RNAs were identified in the media (for full list, see Supplemental 3). Especially in AT-MSC medium, levels of several RNAs were similar to AT-MSC samples. To take this into account, RNAs were required to have at least 2 times higher normalized read count compared to media in all samples to be included in unsupervised clustering and isomiR analysis (see Materials and Methods for details).

### IsomiR identification

**Figure 8:**
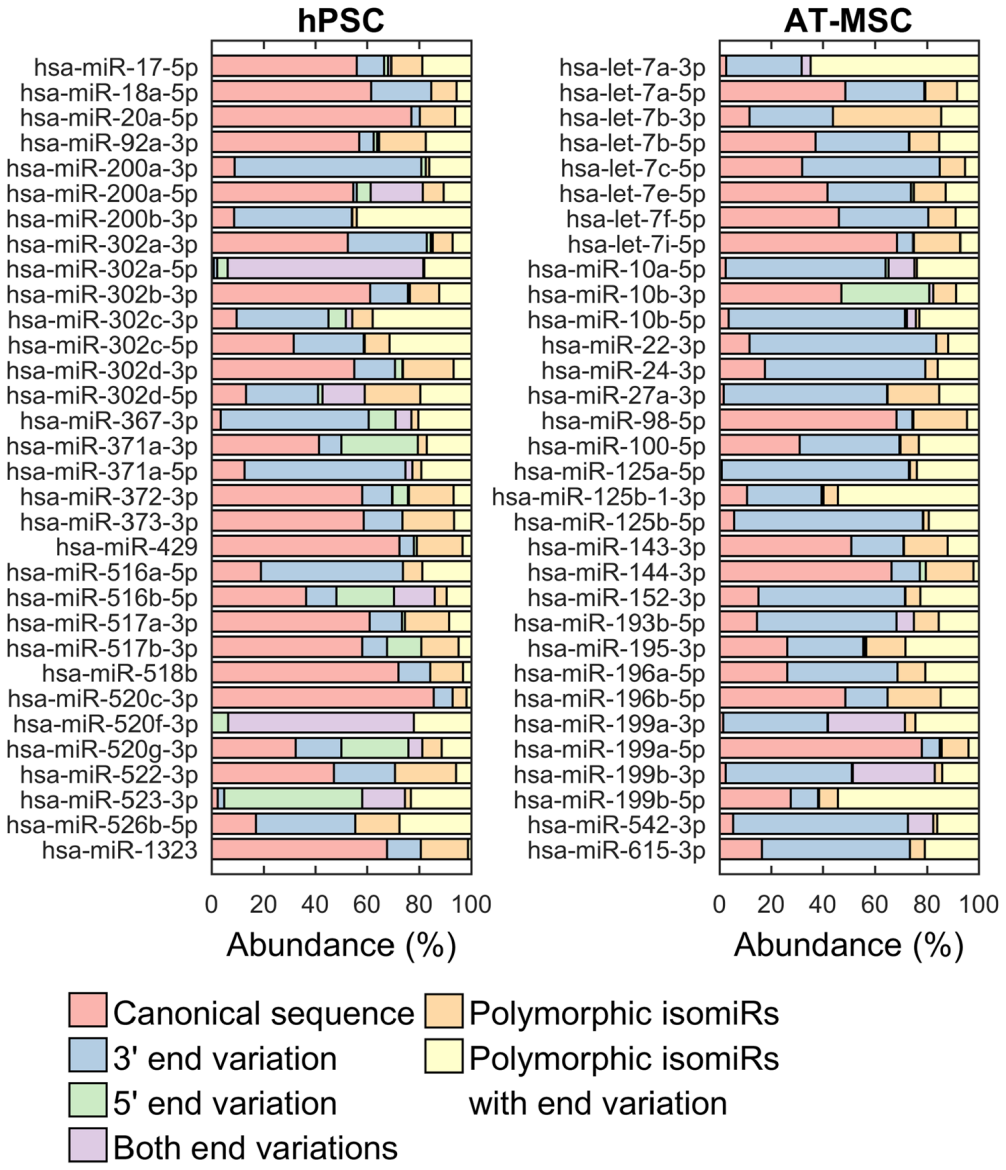
isomiR identification: Distributions of isomiR types in major miRNA families identified in hPSC and AT-MSC EVs.

It is known that majority of miRNA genes encode isomiRs with 3′ or 5′ modifications. We restricted the isomiR identification to the major miRNA families in both stem cell types most abundant miRNAs present in both stem cell types (Tables 1 and 2). Our analysis showed that both hPSC and AT-MSC expressed isomiRs as shown in Fig. 8, where the frequency of different types of modifications in the identified miRNA families are highlighted. Some type of variation was present in a high proportion (72%) of the miRNA reads. In hPSC samples, majority (66%) of miRNAs were mostly comprised of the canonical isomiR and variation in the 3′ end was the most common isomiR type. In contrast, majority (53%) of AT-MSC miRNAs were mostly present as isomiRs with 3′ variation, and only 34% of miRNA were mostly present as the canonical isomiR. However, these common modifications are not as likely to have functional importance as the rarer variations in the 5′ end or in the miRNA seed region. Interestingly, some miRNAs in hPSC EVs (miR-302a-5p, miR-520f-3p, miR-523–3p) were predominantly present as isomiRs with 5′-end modifications.

## Discussion

Understanding the exact composition of EV-derived small ncRNA of defined stem cell type will increase our knowledge about their role in intercellular communication as well as their potential as diagnostic biomarkers. Studies with in-depth characterization of EV-derived ncRNA from clinical grade hPSCs are lacking, and only few studies have explored EV-derived ncRNA of AT-MSCs. The present study utilized high-throughput RNA sequencing to provide an in-depth characterization of small ncRNAs in EVs derived from human AT-MSC and hPSC. This approach identified stem cell specific differences in ncRNA including: miRNA, miRNA isoforms, tRNA, yRNA, snoRNA, and snRNA. Limitation of the study is the low number of samples per group (n = 2–3), as is the case for most sequencing studies on EVs until now. To overcome this, we have reported the miRNAs with the highest expression levels and included seq data from fresh media as controls, to account for the background signal from the media. Some of the miRNA and other small ncRNA identified in our AT-MSC data are in the line with previously published reports, which not only confirm our findings but also indicates that signature EV RNAs are not affected by the donor variations. Our data validated the previously identified cellular genes at EV level, thus highlighting the regulatory potential of EV-ncRNAs and the potential stem cell specific EV biomarkers for future studies.

With all EV isolation methods, a mixed population of vesicles with varying yield is generally obtained. There is no consensus regarding the isolation methods as of date. Therefore, method of choice depends on the downstream applications. Initially, we selected miRCURY EV isolation kit for both EV characterization and EV isolation for RNA sequencing. Despite the dispute about residual cellular RNA in several EV precipitation methods^21^, miRCURY precipitation kit has been reported having better performance. The isolated fraction exhibited specfic surface markers, size distribution of the particles and microRNAs characteristic for EVs^22^. Due to high background with miRCURY precipitation kit, technical problems were encountered at all EV characterization steps. Therefore, we performed EV characterization using ultracentrifugation (UC). In our experience, for EV characterization, isolation of EVs using UC technically stands out as a better option than commercially available precipitation kits^15,23^. Our approach of using two different EV isolation methods may have yielded different and overlapping EV populations. This observation is supported by a recent study where five different EV isolation methods (including UC and precipitation based methods) were compared for profiling miRNAs by sequencing. They concluded that each method captured distinctive but partially overlapping EV populations, along with varying degrees of non-EV contamination^24^.

EV isolation and RNA sequencing was done according to the Exiqon’s workflow. miRCURY precipitation kit was used to purify EVs. Before precipitation, two centrifugation steps were done to remove cellular components and two supernatant removal steps to get as much of the un-precipitated material away before the next step of EVs lysis followed by RNA extraction. Despite these critical attempts to remove non-EV contaminants, we still cannot rule out the possibility that our data is not only based on EV associated RNA, but also RNA co-isolated from the non-vesicular compartment could be also involved.

Small ncRNA profiles of AT-MSCs and hPSCs were unique and highly distinct from each other, which was consistent with previous publications. At transcriptomic and proteomic level, embryonic stem cell and bone marrow stem cells derived EVs displayed different EV-RNA and proteomic profiles^25^. Stem cells and stem cell derived EVs have been reported to exhibit several similar features which affects the stem cell fates, for instance similar miRNA expression profiles between MSCs and MSC-EVs has been documented^26^. In our dataset, 1.4–1.5% of total reads mapped to miRNA in hPSCs and 2.5–4% of reads mapped to miRNA in AT-MSC-2 and AT-MSC-3. However, AT-MSC-1 displayed a different distribution of reads, with markedly greater proportion of miRNA reads (37%). A possible explanation for the discrepancy between AT-MSC-1 and other samples is the gastric bypass surgery and the anti-depression medication used by the donor of AT-MSC-1 sample.

A large fraction of identified EV-miRNAs in our data were also previously reported to be expressed at cellular level^7,26^. miRNAs shuttled by MSC-derived EVs were shown to be involved in the control of transcription, cell survival, multi-organ development, differentiation and immune system regulation^27^.

A low percentage of miRNA reads is typical to extracellular vesicle samples^8,28–33^. Although the number of miRNA reads per sample was relatively low, miRNA reads were of high quality and mostly close to full length (Supplemental 5), as well as showed robust expression in both sample types (Fig. 6).

Several studies have shown that miRNAs are involved in regulating the unique ESC cell cycle, and also the balance between pluripotency and differentiation of hESCs^34–36^. At cellular level, there are generally subtle differences in the miRNA profiles between the two pluripotent cell types (hESCs and hiPSCs)^37^. Our data showed very similar EV-miRNA patterns for the two pluripotent cell types indicating that irrespective of the derivation source, hPSCs release their characteristic miRNA content. In previous studies, human PSCs have shown characteristic miRNA profiles undetectable in adult organs such as miR-302, miR-372, mir-17-92, mir-200, and C19MC families^38^. Our EV-miRNA data showed that hPSC also secreted these aforementioned hPSC specific miRNA clusters (Table 1).

The mir-200 and mir-302 are two large miRNA families involved in maintaining pluripotency by regulating the core regulatory circuitry of pluripotency genes (Nanog, OCT4, SOX2, KLF4). miR-302-driven cellular reprogramming coordinates stem cell division by regulating targets in the cell cycle, particularly at the G1/S restriction point^35,38–40^. Moreover miR-302 and miR-372 cluster miRNAs promote human somatic cell reprogramming to iPSCs^41,42^. Furthermore, these miRNAs repress multiple target genes regulating various other cellular processes, including epithelial-mesenchymal transition (EMT), epigenetic regulation and vesicular transport.

The chromosome 19 miRNA cluster (C19MC) is one of the largest miRNA gene clusters in the human genome. Expression of mir-516a, b-5p, 517a, b-3p, 518b, 520c, f, g-3p, 522-3p, 523-3p, 526b-5p, 1323 from C19MC was detected in our analysis. This primate-specific miRNA cluster is restrictively expressed in pluripotent ESCs^40,43,44^ and, later during embryonic and fetal development, only in the placenta but not in other adult organs and tissues^45^. C19MC miRNAs represent the majority of miRNAs not only in the trophoblast but also in EVs derived from it. The target genes are strongly associated with developmental processes and various cancers. It is suggested that these miRNAs may play critical roles in regulating differentiation and growth during the early development and in maintaining the pluripotency of hESCs^46^.

ESC-EVs have shown to promote the survival and improve the expansion of hematopoietic progenitor cells^47^ and to induce de-differentiation and alterations in gene expression on cultured retinal progenitor Müller cells^48^. EV-treated Müller cells showed up-regulation of genes and miRNAs associated with cellular proliferation and induction of pluripotency and down-regulation of genes important to differentiation and cell cycle arrest. These findings suggest that by transferring stem cell-specific molecules, EVs can induce the activation of endogenous, adult, quiescent progenitor cells, increasing their pluripotency and possibly their ability to repair damaged tissues. Our data confirmed that PSC-specific miRNA previously identified on cellular level are also found in EVs. Furthermore, additional EV-specific miRNAs were also identified, but deciphering their role need further studies.

In order to establish a global miRNA expression profile of MSCs, the consensus miRNAs in MSCs detected by different methods and from various sources have been previously reported^49^. Among which mir-199a, 152, 125a, b, 143,100 and let-7b, c, e, f, were also identified in our AT-MSCs derived EVs (Table 2). Altogether our results strengthen the hypothesis that signature MSC miRNA are also expressed in their EV cargoes. Nine out of the highly expressed EV-miRNAs in our AT-MSC samples (let-7a-5p, let-7b-5p, let-7i-5p, miR-125-5p, miR-199a-3p, miR-199b-3p, miR-100-5p, miR-144-3p and miR-22-3p) have previously been also found to be present in bone-marrow-derived MSC EVs^50^. This clearly highlights that independent of cell of origin, MSCs share certain characteristic miRNA-EVs which are involved in regulating differentiation. mir-10a-5p, mir-10b-5p, mir-22-3p, mir-143-3p, mir-100-5p, let-7a, f-5p identified in our data have also previously been listed as most abundant miRNA in AT-MSCs EVs^12^. These results indicate that expression of these miRNA is unaffected by donor variability.

There are substantial numbers of experimental data confirming that miRNAs have multiple roles in regulating bone remodeling^51^ as well as osteogenesis. miRNAs are known to regulate osteoblast differentiation positively by targeting negative regulators of osteogenesis or negatively by targeting osteogenic factors^52^. Recently it has been reported that MSCs secrete miRNA and other small RNAs via EVs^12^. Our data revealed that in AT-MSCs, EV-miRNAs that modulate osteogenic commitments of AT-MSC, for example; let-7a and c, mir-22, 199a, 196a, 199b and negative regulators of osteogenic differentiation such as mir-27, 98, 100, 615, 125b and 195 were enriched exclusively in AT-MSCs as compared to hPSCs (Table 2). This indicates that these EV-miRNAs have the potential to modulate bone differentiation pathways of the target cell.

Specifically, *let-7* family, which is classically involved in stem cell differentiation, is considered as a global regulator of differentiation^53^. *let-7c* is known to enhance osteogenesis and thus bone formation while repressing adipogenesis of human MSCs by targeting HMGA2^54^. let-7a and mir-199b were detected in the EVs during osteogenic differentiation of BM-MSCs^55^. miR-199b-5p displayed positive role in osteoblast differentiation as it was significantly up-regulated during the osteogenesis in human BM-MSCs. Its functions as a positive regulator of osteogenesis is likely due to its involvement in GSK-3β/β-catenin signaling pathway^56^. miR-199a has been shown to increase osteogenic differentiation *in vitro* and to enhance ectopic bone formation *in vivo*. *E*xpression of this miRNA is controlled by a HIF1α and Twist1 cyclic pathway^57^. mir-22 has been reported to enhance osteogenic differentiation of AT-MSCs by repressing its target *HDAC6* and also by acting as a crucial regulator of switch between adipogenic and osteogenic differentiation^58^. EV delivery significantly enhances bone formation *in vivo* and bone regeneration has been speculated to be regulated by EV-derived mir-196a^59^. This miRNA has been shown to inhibit proliferation and enhance the differentiation process by regulating HOXC8^60^.

Negative regulators of osteogenic differentiation (miR-100-5p, miR-125b-1-3p, miR-98-5p, miR-195-3p, miR-24-3p, miR-27a-3p, and miR-615-3p) were exclusively expressed in our AT-MSC samples as opposed to hPSC samples. Many of these miRNAs affect well-known BMP induced osteogenic differentiation^61^. In AT-MSCs overexpression of miR-100 inhibited osteogenic differentiation of stem cells by targeting BMPR2^62^. mir125b directly targeting BMPR1b was identified as negative regulator of osteogenic differentiation of human BM-MSC^63^. mir-98 in BM-MSCs regulated osteogenic differentiation by targeting BMP2^64^. mir-195 not only acts as a negative regulator of osteogenesis in human BM-MSCs, but also as an inhibitor of stem cell proliferative capacity and as an anti-angiogenic by targeting VEGF^65^. mir-27, whose promoter is negatively regulated by RUNX2, functionally inhibits osteogenesis by downregulating SATB2, which is an important regulator of osteogenic differentiation^66^. mir-615 negatively regulates the osteogenic differentiation by suppressing osteogenic regulators GDF5 and FOXO1^67^. Thus EV-miRNAs which are negative regulators of osteogenesis could be the potential targets for enhancing bone regeneration as they can by directly delivered as anti-mir oligonucleotides into bone injury sites. The approach of using EVs as vehicles to deliver miRNA mimics or anti-mirs is certainly a potential tool in future bone regeneration applications.

IsomiRs, which are functionally different from their canonical microRNA are most probably generated by variation in processing by Drosha and*/*or Dicer and differ by variations of a few bases at 5′ and 3′ end of miRNA^68,69^. They affect the half-life of miRNA, their sub-cellular localization and also their target specification^70–72^. IsomiRs with variations at the 3′ end were the predominant isomiR category identified in our data in both stem cell types (Fig. 8), and the ratio of isomiR reads to canonical miRNA reads was similar to earlier studies^33,73^. Exact function of isomiRs with 3′ modifications are not clearly known but are suggested to be related to a disease state^74^. In hPSC for example, we found that miR-302a, an important miRNA for hESCs self-renewal, showed predominant expression of the isomiR species with 5′ –end variations over its canonical miRNA. mir-302a isomiRs with unique seed sequences have indicated specificity in their target selection and therefore likely to be significant regulators of cellular differentiation^75^. Importance of the isomiRs in different stem cell types with variations at 3′ and 5′ end were reported by Tan *et al*.^69^, indicating that isomiRs are active *in vivo* and thereby have functionally importance as they co-immunoprecipitate with Ago proteins and are active in *in vitro* luciferace and cleavage assays.

Apart from miRNAs, 16 snRNA and 67 snoRNAs were the most highly-expressed non-coding RNA in hPSC-EVs. These non-coding RNAs play important roles in mRNA biogenesis and maturation^8,76^ and are also known to be involved in human cancers^77^. ncRNAs such as y and tRNA fragments displayed selectivity for AT-MSC which was consistent with recent reports^12^. Recent studies have reported that Y RNA fragments may play role in histone mRNA processing and cell damage^78,79^ and DNA replication^80^. Fragments derived from tRNA play important roles in regulating many biological processes, such as reverse transcription and guidance of other RNAs^81^. Functions of these ncRNAs in stem cell biology needs to be further elucidated.

Fetal bovine serum (FBS) used in cell culture experiments contains bovine EVs which interferes with the EVs produced by the cells. In our previous work we have reported that FBS used in cell culture experiments contains abundant amounts of EVs^15^. Ultracentrifugation (100,000 g for 19 hours), the most commonly used protocol for removal of FBS derived EVs, does not completely remove the EVs^15^. The current study support our previous findings, confirming presence of FBS derived miRNAs and small ncRNA in the EV depleted media (Fig. 7). Specifically, mir-122-5p, mir-423-5p and mir-148a-3p were the abundant miRNAs present in the media. mir-122 has also been previously reported to be highly enriched in FBS^82^. Other miRNA and small ncRNA present in EV depleted FBS are shown in the Supplemental 3. This issue should be taken into consideration when drawing final conclusions about cell culture results as the interference and the biological effects associated with FBS derived RNA is not clearly known. hPSC medium used for culturing hPSCs, as expected, showed insignificant amounts of miRNAs and ncRNAs, assumed to be derived from medium components.

## Conclusion

Overall, in our small scale analysis, we found that both AT-MSCs and hPSCs secrete a selective pattern of small ncRNA in there cell-free secretome. Their transfer to the target cells could be a mechanism of action for maintaining the stem cell specific characteristics, orchestrating gene expression, and to mediating communication between neighboring cells. Understanding of paracrine regulation of pluripotency and differentiation commitment of stem cells, could have implications on the improvement of stem cell cultures and differentiation protocols. Thus, in-depth understanding of EV-derived ncRNA regulatory mechanisms could provide strategies for developing engineered EVs with therapeutic RNA.

## Material and Methods

### Culture and characterization of stem cells

#### hPSC lines

Human ESC line hPSC-1 was derived from surplus, blastocyst stage human embryo as described previously^83^. Human iPSC line hPSC-2 was generated at Prof. Katriina Aalto-Setälä’s laboratory at University of Tampere from dermal fibroblasts (Table 3). For this study, hPSCs were cultured in xeno- and serum-free Essential 8™ Flex Medium (E8 flex, Thermo Fisher Scientific), supplemented with 50 U/ml Penicillin-Streptomycin (Gibco, Thermo Fisher Scientific) on Corning® CellBIND® -well plates coated with 0.55 µg/cm^2^ human recombinant laminin-521 (LN-521, Biolamina, Sweden). Single cell passaging with TrypLE™ Select Enzyme (Gibco, Thermo Fisher Scientific) was carried out twice a week using split ratio of 40 000-50 000 cells/cm^2^ as described by Hongisto *et al*.^84^.

**Table 3:** Details of the hPSC and AT-MSC cell lines used in this study.

Cell line ID	Sex/Karyotype	Passage	Source/Details	BMI	AGE
hPSC-1	46XX	p28 + 9FF	Blastocyst	—	—
hPSC-2	46XX	p26 + 9FF	Skin/Sendai	—	—
AT-MSC-1	XX	P5	Thigh	23.6	53
AT-MSC-2	XX	P4	Abdominal flanks	22.7	32
AT-MSC-3	XX	P1	Thigh	31.2	50

#### hPSC characterization

Human PSCs were characterized as described previously^84^. Briefly, the hPSCs were monitored regularly microscopically, and characterized by immunofluorescence staining (IHC) for expression of pluripotency markers using the following primary antibodies; Nanog (1:200, R&D Systems, AF1997), OCT-3/4 (1:200, R&D Systems, AF1759), SSEA-3 (1:300, Novus Biologicals NB100–1832), SSEA-4 (1:200, R&D Systems, MAB1435), TRA-1-60 (1:200, Millipore, MAB4360), TRA-1-81 (1:200, Santa Cruz Biotechnology SC-21706), and early marker for differentiation SSEA-1 (1:200, Santa Cruz Biotechnology, SC-21702). Alexa Fluor-conjugated (1:400, ThermoFisher Scientific A-11055, A-21042, A-10037), and FITC-conjugated (1:400, Novus Biologicals, NB7102) secondary antibodies were used. Nuclei were counterstained with 4′,6′diamidino-2-phenylidole (DAPI) (Vector Laboratories Inc., Burlingame, CA).

Pluripotency was verified with *in vitro* pluripotency assay by spontaneous differentiation as embryonic bodies^84^, followed by immunofluorescence analysis for alpha-smooth muscle actin (SMA, 1:400, R&D Systems, MAB1420) for mesoderm, alpha-fetoprotein (AFP, 1:200, R&D Systems MAB1369) for endoderm, and OTX2 (1:200, R&D Systems, AF1979) for ectoderm. Karyotyping was performed at Finnish Microarray and Sequencing Centre (FMSC), Turku Centre for Biotechnology with the KaryoLite BoBs assay (Perkin Elmer).

#### AT-MSC lines

Human AT-MSC were obtained from water jet-assisted liposuction aspirates^85^ from three donors using mechanical and enzymatic isolation as described previously^86^. All donors were female, age range of 32–53 (average 45) and BMI range of 22.7–31.2 (average 25.8) (Table 3). Cells were cultured in AT-MSC media consisting of Dulbecco’s modified Eagle’s medium/Ham’s Nutrient Mixture F-12 with 1% L-alanyl-L-glutamine (DMEM/F-12 1:1 GlutaMAX; Gibco ref. 31331–028, lot. 1765999), 1% antibiotics (100 U/ml penicillin, 0.1 mg/ml streptomycin; Lonza ref. DE 17–602 E, lot. 5MB 068) and 10% FBS (Fetal Bovine Serum, South American ref. 10270106, lot. 42F8554K) at 37 °C and 5% CO_2_^15^. Once AT-MSCs had adhered to the culture flask, non-adherent populations were gently washed away with PBS and fresh culture media was added.

#### AT-MSC characterization

Cells cultured in AT-MSC media (n = 3) were characterized using BD Accuri C6 flow cytometer (Becton Dickinson, Franklin Lakes, NJ, USA) to confirm the mesenchymal origin of the cells. Allophycocyanin (APC)-conjugated monoclonal antibodies against CD14 (clone: M5E2), CD19 (clone: HIB19), CD34 (clone: 581), CD45RO (clone: UCHL1), CD54 (clone: HA58), CD73 (clone: AD2), CD90 (clone: 5E10), CD105 (clone: 266) and HLA-DR (clone: G46-6) (BD Pharmingen, Becton Dickinson) were used. Analysis was performed on 100.000 cells per sample, and the positive expression was defined as the level of fluorescence greater than 99% of the corresponding unstained cell sample^15^. Flow cytometric data is shown as average with standard deviation. Cells from three donors were plated at 2.000 cells/cm^2^ in four replicates per condition in 24-multiwell plate to analyse the osteogenic potential of AT-MSCs. Cells were first plated in AT-MSC media. After 24 h, osteogenic differentiation was induced using osteogenic media (OM; AT-MSC media supplemented with 50 µM L-ascorbic acid 2-phosphate, 10 mM β-glycerophosphate disodium salt hydrate and 5 nM dexamethasone (all Sigma-Aldrich). At 7, 14 and 21days time points, osteogenesis was assessed using quantitative alkaline phosphatase activity (qALP) analysis which was normalized with total DNA quantification and quantitative Alizarin red staining (qARS) as reported previously^15,87^.

### EV isolation and basic characterization

hPSC media collection for EV extraction was performed from four consecutive passages for both cell lines. Media were collected from 80–100% confluent cells cultures. The day after passaging, the hPSCs were rinsed twice with DPBS (Lonza), and fresh hPSC medium was added to the cells. 48 hours later, the hPSC conditioned media were collected to 50 ml Falcon tube, and immediately centrifuged 2,000 × g for 10 minutes at 4 °C to remove cell debris. Supernatant was transferred to a new Falcon tube and frozen to -80 °C for storage. Non-conditioned hPSC medium was collected as control. Prior to EV isolation, AT-MSCs were grown in EV-depleted medium for 72 hours. EV-depletion was performed as described previously^15^. Briefly, EV depleted FBS (dFBS) was prepared by 19 h ultracentrifugation of regular FBS at 26 000 rpm (121 896 gmax) using an SW28 rotor (k-factor 284.7, Beckmann-Coulter). Only the light coloured top layers of the supernatant (approx. 9/10) were retained and used in the subsequent analyses. The dFBS was filtered with a 0.22 μm filter (Millipore Stericup-GP, 0.22 μm, polyethersulfone filter) before addition to cell-culture medium. For characterization, EVs were isolated from conditioned culture medium using ultracentrifugation as described previously^15^. Briefly, the conditioned medium was depleted of cell debris by centrifuging for 10 min at 2,500 x g and only the AT-MSC supernatant was filtered through a 0.45 μm sterile filter (Merck Millipore). EVs were extracted using ultracentrifuge at 26,000 rpm (121 896 gmax) for 2 hours at 4 °C with SW28 rotor to collect the EV pellet, which was washed by filtered PBS and stored in Protein LoBind microcentrifuge tubes (Eppendorf) at −80 °C. For RNA sequencing, EVs were isolated from the conditioned medium by precipitation using the miRCURY^TM^ Exosome Isolation Kit (Exiqon A/S, Vedbaek, Denmark) according to the manufacturer’s instructions.

### Nanoparticle tracking analysis (NTA)

The number and size distribution of particles from isolated EV samples were analysed using Nanosight model LM14 (NanoSight Technology, Salisbury, U.K., http://www.malvern.com) equipped with blue (404 nm, 70 mW) laser and CMOS camera (Hamamatsu Photonics K.K., Hamamatsu City, Japan). For the analyses samples were diluted in filtered (0.1 µm) DPBS to obtain the optimal detection concentration of 10^6^–10^9^ particles/ml, and triplicate 60 s videos were recorded using camera level 13. The data was analysed using NTA software 3.0 with the detection threshold 5^88^.

### Transmission electron microscopy

Particle morphology was examined using transmission electron microscopy (TEM) Tecnai 12 (FEI Company, Eindhoven, the Netherlands) operating at 80 kV as described previously^89^. Briefly, after loading to 200 mesh copper grids and fixation with 2% PFA in 0.1 M NaPO_4_ buffer (pH 7.0), samples were washed with the 0.1 M NaPO_4_ buffer and deionized water, negatively stained with 2% neutral uranyl acetate and embedded in methyl cellulose uranyl acetate mixture (1.8/0.4%). Images were taken with Gatan Orius SC 1000B CCD-camera (Gatan Inc., USA) with 4008 × 2672 px image size and no binning. Samples from 2–3 biological replicates were viewed.

### Western blotting

Western blotting was performed as described previously^89^ using primary antibodies against Hsp70 (#554243, BD Biosciences), and CD63 (#556019, BD Biosciences) at 1:1000 dilution, as well as TSG101 (#SAB2702167, Sigma-Aldrich), and CD90 (#WH0007070M1, Sigma-Aldrich) at 1:500 dilution. The lack of calnexin served as an indicator for the purity of EVs samples, calnexin is an endoplasmic reticulum protein; probing of such protein involved the use of Calnexin (C5C9) Rabbit mAb (#2679, Cell Signaling Technology) at 1:800 dilution. EVs isolated by ultracentrifugation from equal volumes (20 mL) of each sample were loaded to gels. As controls, 30 µg of protein from AT-MSC and hPSC lysates measured by BCA assay (Pierce BCA Protein Assay Kit) were used, growth media for both AT-MSC and hPSC served as negative control samples. Probing for CD63 and CD90 was done on individual samples, however, due to limited amount of EV samples, Hsp70 and TSG101 protein detection was detected from pooled samples. Samples were denatured at 95 °C for 5 min in reducing Laemmli sample buffer; except for CD63 detection which was run in non-reducing conditions, separated using Mini-PROTEAN^®^ TGX™ 12% gradient SDS-PAGE gel (Bio-Rad, Hercules, CA, USA) with prestained protein ladder (BlueSTAR Prestained Protein Marker, # MWP03, Nippon Genetics Europe GmbH) as a standard, running conditions were 150 V for 60 minutes. Blotting involved semi-dry transfer of proteins on nitrocellulose membranes, 0.2 µm (#162–0112, BIORAD), using 40 mA per gel for 60 minutes. Blocking and antibody incubations were performed in Odyssey blocking buffer (LI-COR) with and without 0.1% Tween-20. After primary antibody overnight incubation at +4 °C, membranes were washed 4 × 5 minutes in TBS-T, and probed with secondary IRDye^®^ 800CW Goat (Li-COR) at 1: 15,000 for 1 hour at RT. After incubation, membranes were washed 4 × 5 minutes in TBS-T at RT and briefly rinsed with PBS 1×, then imaged on an Odyssey FC Imager (Li-COR). All original WB images are shown in Supplemental 4.

### Small RNA sequencing

Sequencing experiments for three AT-MSC samples and two hPSC samples and the corresponding pure media were conducted at Exiqon Services, Denmark. EVs were isolated from 3 ml conditioned cell culture media using miRCURY™ Exosome Isolation kit (Exiqon A/S). Before precipitation, two centrifugation steps were used to remove cellular components and two supernantant removal steps to get rid of the un-precipitated material, followed by EV lysis and RNA isolation using miRCURY™ RNA Isolation Kits - Cell & Plant (Exiqon A/S). Good performance of Exiqons EV isolation kit has been reported^22^. NGS libraries were prepared using the NEBNext® Small RNA Library preparation kit (New England Biolabs), consisting of adapter ligation, cDNA conversion, PCR amplification (18 cycles) and purification. From a total of 50 µl isolated RNA, 6 μl was converted into microRNA NGS libraries. Library preparation QC was performed using Bioanalyzer 2100 (Agilent). Based on the quality of the inserts and the concentration measurements the libraries were pooled in equimolar ratios. The pool was then size selected using the LabChip XT (PerkinElmer) aiming to select the fraction with the size corresponding to microRNA libraries (~145 nt). The library pools were quantified using the qPCR KAPA Library Quantification Kit (KAPA Biosystems). Single-ended sequencing with 50 cycles was then performed on the library pools using a NextSeq500 sequencing instrument according to the manufacturer instructions. Raw data was de-multiplexed and FASTQ files for each sample were generated using the bcl2fastq software (Illumina inc.).

Reads were mapped using Bowtie 2 software^90^ according to the Exiqon NGS Service microRNA/smallRNA pipeline. First, reads mapped to spike-ins or outmapped (mapped to adapter sequences, ribosomal RNA, ΦX174-genome, mitochondrial RNA, or polyA and polyC homopolymers) with the following parameters: ‘*-N 0 -L 32–no-1mm-upfront -R 10 -D 15–n-ceil C*,*0–score-min C*,*0*’, were discarded. Second, reads were mapped to mature miRNAs from miRBase 20 with the same parameters. Third, unaligned reads were mapped to GRCh37 genome with the parameters ‘*-N 1 -L 32 -R 4 -D 20–n-ceil L*,*0*,*0*.*15–rdg 200*,*51–rfg 200*,*51–score-min C*,*-250*’. The reads were then quantified (including isomiR quantification) using custom Ruby scripts. IsomiRs were defined as sequences with a single base substitution or having 5′ and 3′ insertion/deletion compared to the mature canonical miRNA sequence.

Only RNAs with more than 1 CPM in at least two samples were included in the analysis. Unsupervised clustering was performed using Euclidean distance metric and average linkage. Differential expression was calculated using the exact test for negative-binomially distributed counts with tagwise dispersion in the edgeR package 3.20.1^91^. For a large proportion of the identified miRNAs and smRNAs, similar quantities were also detected in the corresponding unconditioned media, especially for AT-MSC derived EVs (Fig. 8C,D), but also for hPSC EVs (Fig. 8A,B). Therefore, to filter out the RNAs possibly originating from the media, the following procedure was employed: first, raw read counts were normalized to external spike-ins (UniSp100-UniSp151), since assuming same levels of RNA for unconditioned media and EV samples is not appropriate. Second, RNAs overexpressed in either sample group (|Fold change| > 2) were required to have at least 2-fold higher spike-in normalized counts in all samples than in the corresponding media to be included in the analyses. RNAs with similar expression levels between samples (|Fold change| < 2) were required to have at least 2-fold normalized counts in both sample groups. Multiple hypothesis correction was performed using the Benjamini-Hochberg procedure^92^ with a FDR limit of 0.01.

We have submitted all relevant data of our EV characterization to the EV-TRACK knowledgebase (EV-TRACK ID: EV180022)^93^_._

#### Ethical approval and informed consent

For the hPSCs, the research groups at the University of Tampere have approval of the National Authority for Medicolegal Affairs Finland (Dnro 1426/32/300/05) to use human embryos for research purposes, and supportive statements of the Ethical Committee of the Pirkanmaa Hospital District to derive, culture, and differentiate hESC lines (Skottman/R05116) and to use hiPSC lines in ophthalmic research (Skottman/R14023). No new hPSC lines were derived for this study.

For the AT-MSCs, the study was carried out with supportive statements of the ethical committee of Helsinki and Uusimaa Hospital District for the use of adipose tissue and derivatives (DNro 217/13/03/02/2015) and with informed consent from the donors. All methods were carried out in accordance with the relevant guidelines and regulations.

## Electronic supplementary material

Supplemental 1,4 and 5

Supplemental dataset 2 and 3

## Data Availability

The RNA sequencing data has been deposited to the GEO (accession number GSE113868). We have submitted all relevant data of our EV characterization to the EV-TRACK knowledgebase (EV-TRACK ID: EV180022).
