# Supplementary material for: Small non-coding RNA landscape of extracellular vesicles from human stem cells
Source: Sci Rep. 2018 Oct 19;8:15503. doi: 10.1038/s41598-018-33899-6 (PMC6195565; doi:10.1038/s41598-018-33899-6)

Small non-coding RNA landscape of extracellular vesicles from human stem cells

Sippy Kaur, Ahmed G Abu-Shahba, Riku O Paananen, Heidi Hongisto, Hanna Hiidenmaa, Heli Skottman, Riitta Seppänen-Kaijansinkko, Bettina Mannerström

Supplemental 1 Characterisation for human PSC-2. **A)** Human PSC-1 characterized for A) typical undifferentiated colony morphology in phase contrast image and **B)** expression of pluripotency markers Nanog, OCT-3/4, SSEA-3, SSEA-4, TRA-1-60, and TRA-1-81, and lack of expression of early differentiation marker SSEA-1 after immunofluorescence staining. Corresponding nuclei stains with DAPI shown. **C)** The results of the KaryoLite BoBs assay are shown as signal relative to karyotypically normal female (/F, red) and male (/M, blue) genomic DNA used as a reference (equal to 1) for each of the probes covering both p and q arms of all chromosomes. Software threshold for changes shown as a green lines and deviations in red. **D)** Pluripotency shown after spontaneous differentiation *in vitro* as expression of markers for mesoderm, endoderm, and ectoderm. All scale bars 200 µm.

A

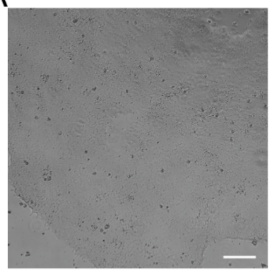

B

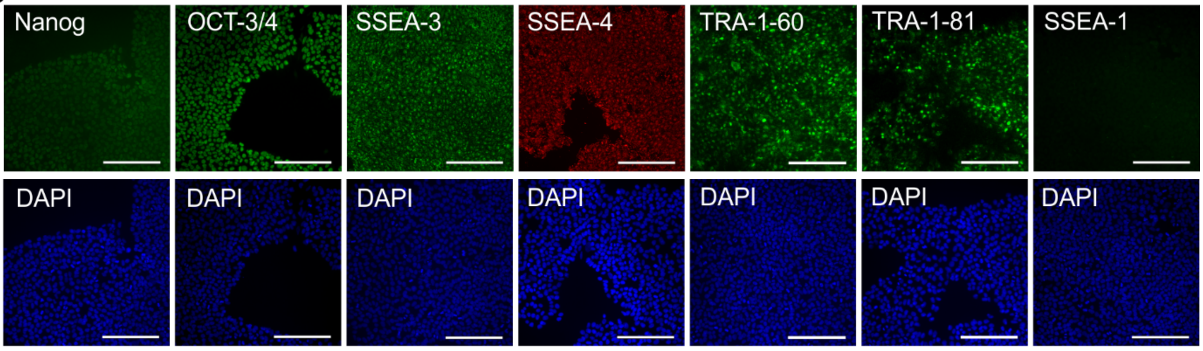

C

Normalized Ratios

Probe	/F	/M	Chromosomal Location
01C	0.96	0.98	
02C	0.98	1.01	
03C	1.02	1.02	
04C	1.02	1.00	
05C	0.92	0.94	
06C	0.95	0.96	
07C	0.94	0.99	
08C	0.97	0.97	
09C	1.02	1.04	
10C	1.01	1.01	
11C	1.03	1.03	
12C	1.03	1.01	
13C	1.07	1.09	
14C	0.96	0.94	
15C	1.02	1.01	
16C	0.98	1.04	
17C	1.01	1.00	
18C	0.94	1.00	
19C	1.04	1.06	
20C	1.05	1.01	
21C	1.05	1.01	
22C	1.04	1.06	
Auto	0.99	0.99	
X1;ptel	0.99	1.46	-
X2;pcen	0.96	1.55	-
X3;qcen	0.96	1.37	-
X4;qtel	0.97	1.43	-
Y1;ptel	1.01	0.61	-
Y2;pcen	0.90	0.33	-
Y3;qcen	0.93	0.67	-
Y4;qtel	0.85	0.37	-

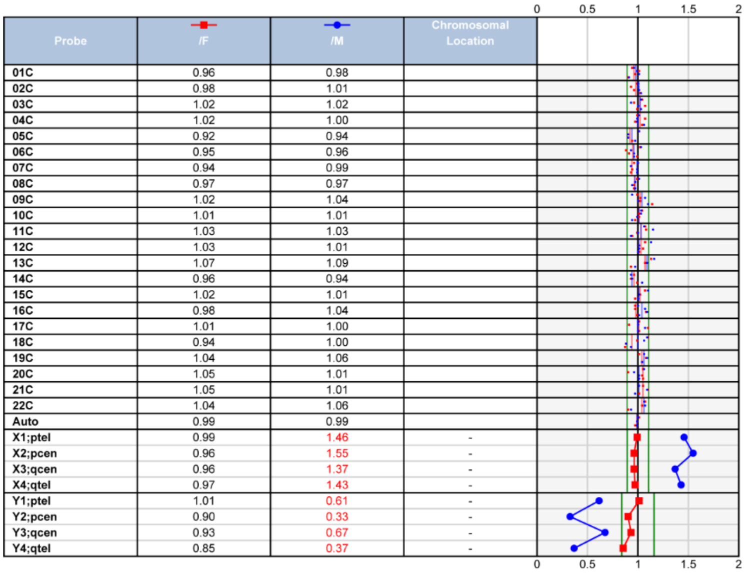

D

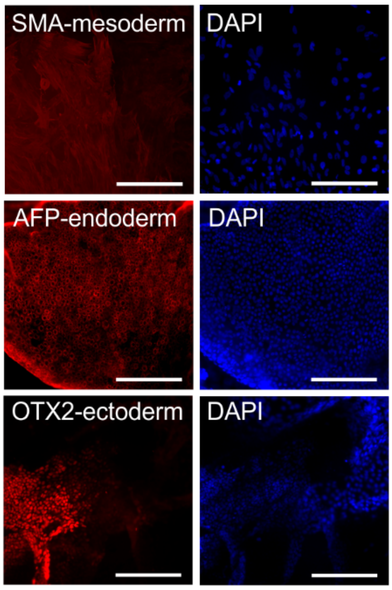

Supplemental 4 Original western blot images for all samples. Samples unmarked and unrelated to this project are also in blots for CD90, HSP70 and TSG101.

Calnexin

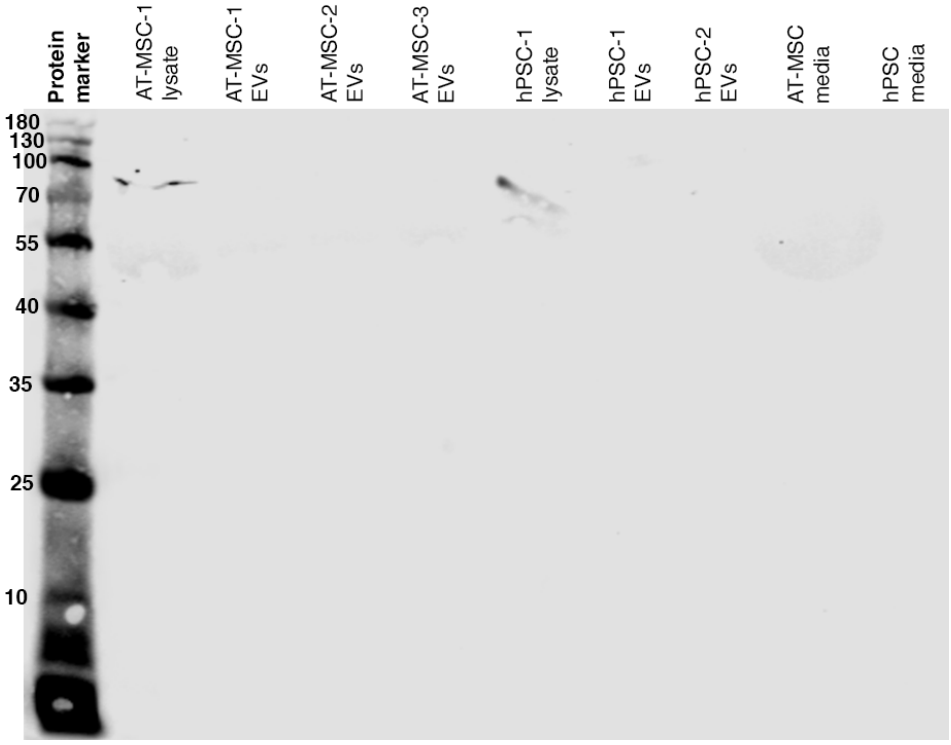

CD63

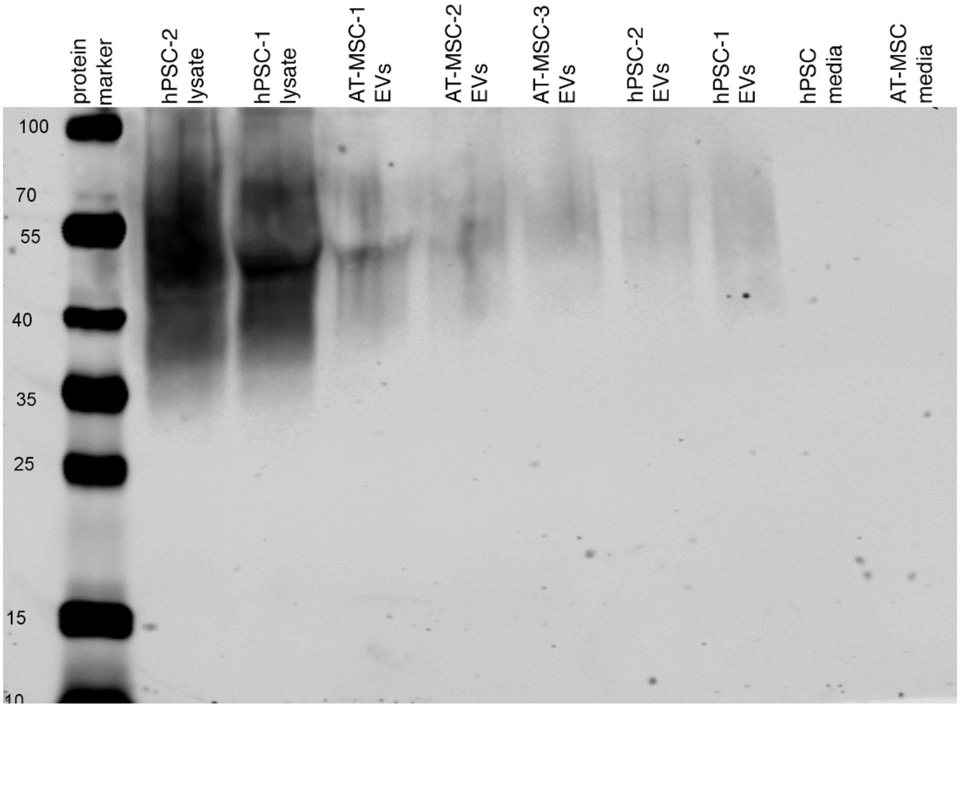

CD90

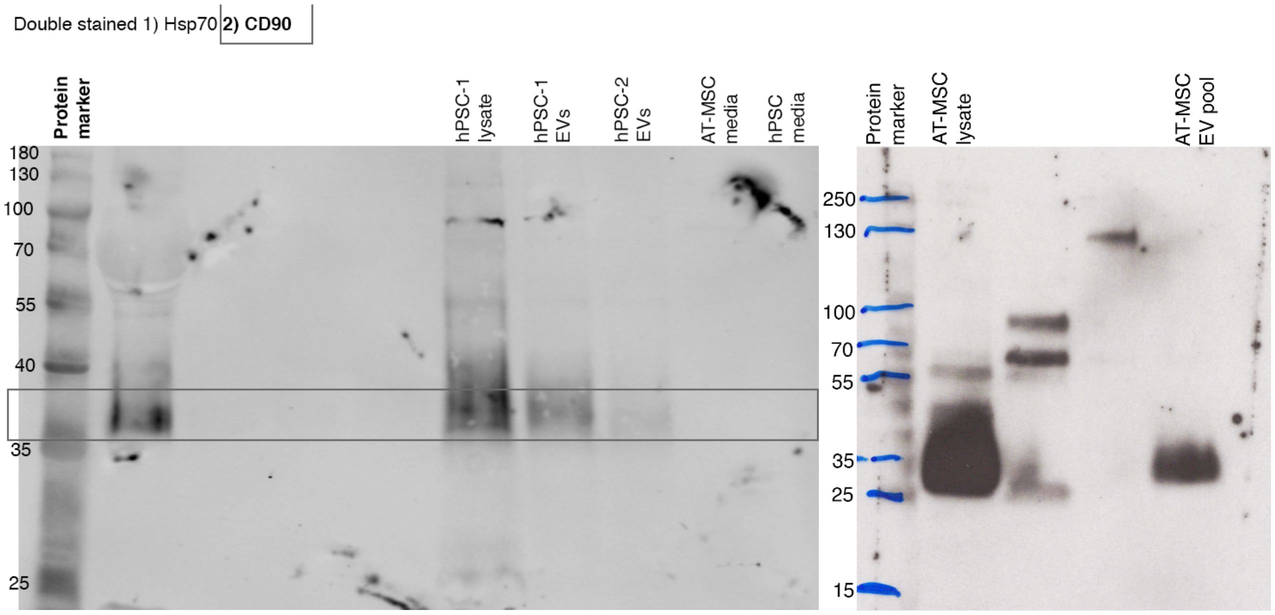

Double stained 1) Hsp70 2) CD90
Protein marker
hPSC-1 lysate
hPSC-1 EVs
hPSC-2 EVs
AT-MSC media
hPSC media
180
130
100
70
55
40
35
25
Protein marker
AT-MSC lysate
AT-MSC EV pool
250
130
100
70
55
35
25
15

Hsp70

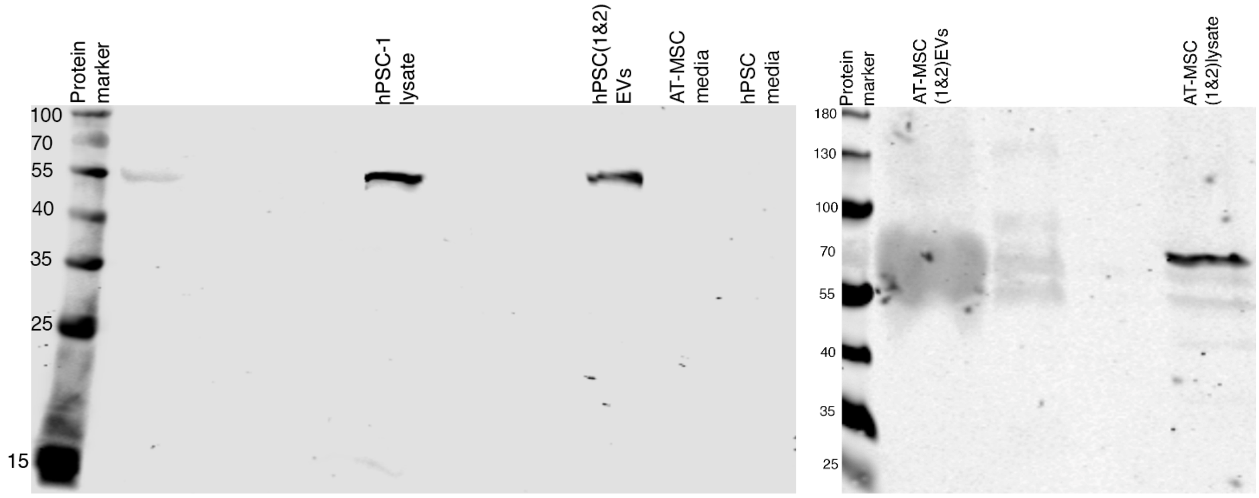

Protein marker
hPSC-1 lysate
hPSC(1&2) EVs
AT-MSC media
hPSC media
100
70
55
40
35
25
15
Protein marker
AT-MSC (1&2)EVs
AT-MSC (1&2)lysate
180
130
100
70
55
40
35
25

TSG101

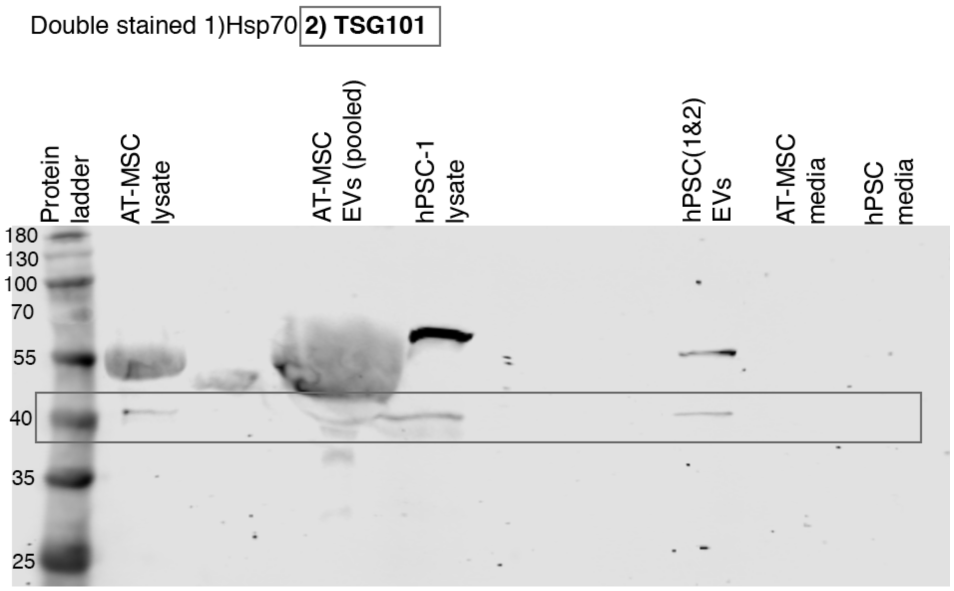

Supplemental 5 miRNA Phred quality scores and insert length distributions for all the samples in the study.

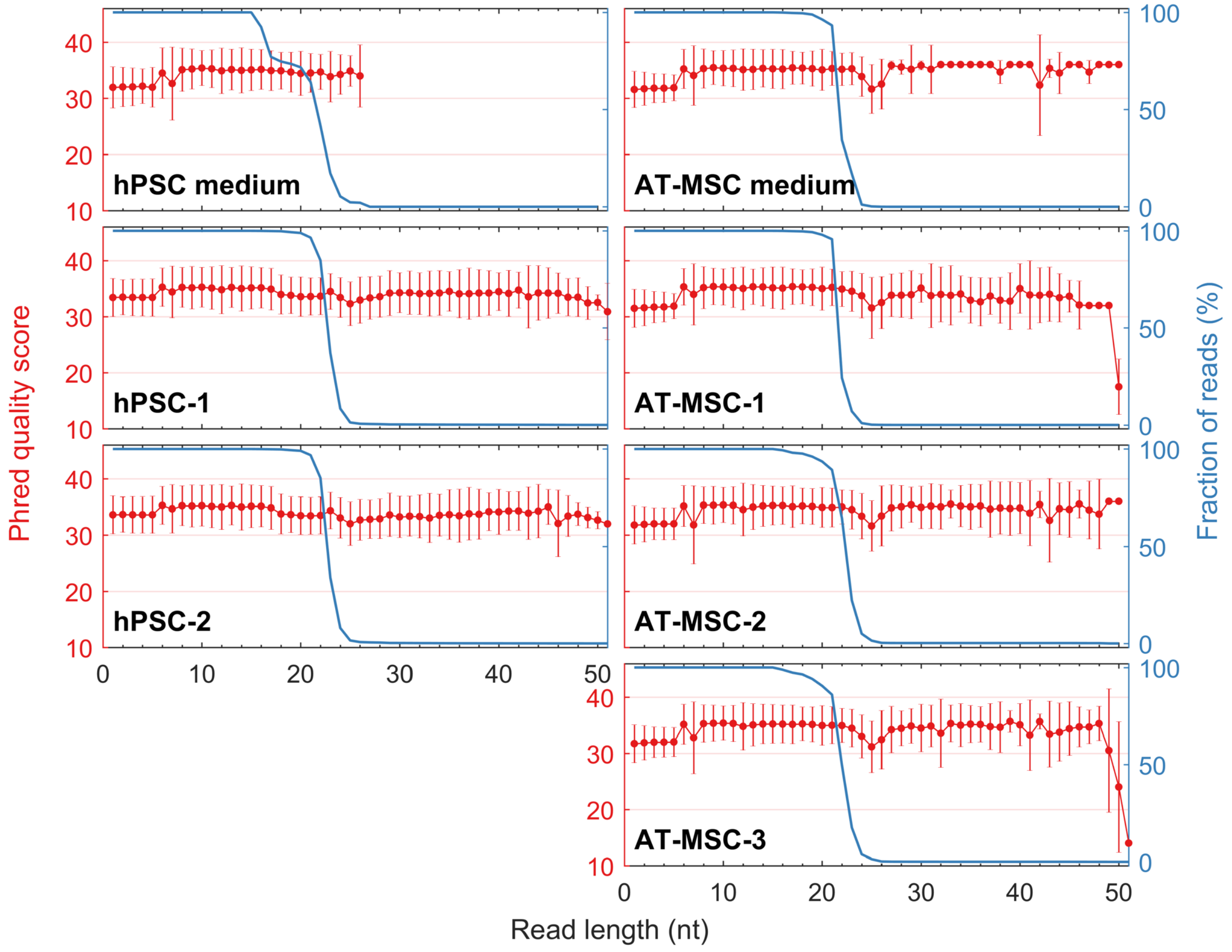

Supplement: Supplementary file 1 — Supplemental 1,4 and 5 [file 41598_2018_33899_MOESM1_ESM.pdf]
